# Using qualitative research methods in biomedical innovation: the case of cultured red blood cells for transfusion

**DOI:** 10.1186/s13104-016-2077-4

**Published:** 2016-05-11

**Authors:** Catherine Lyall, Emma King

**Affiliations:** Science, Technology and Innovation Studies, School of Social and Political Science, The University of Edinburgh, Edinburgh, UK; NMAHP Research Unit, University of Stirling, Stirling, UK

**Keywords:** Cultured red blood cells, Public engagement, Participatory research, Interdisciplinarity, Focus groups, Interviews, Qualitative research, Upstream engagement

## Abstract

**Background:**

Qualitative research has a key role to play in biomedical innovation projects. This article focuses on the appropriate use of robust social science methodologies (primarily focus group studies) for identifying the public’s willingness and preference for emerging medical technologies. Our study was part of the BloodPharma project (now known as the Novosang project) to deliver industrially generated red blood cells for transfusion. Previous work on blood substitutes shows that the public prefers donated human blood. However, no research has been conducted concerning attitudes to stem cell derived red blood cells.

**Method:**

Qualitative research methods including interviews and focus groups provide the methodological context for this paper.

**Results:**

Focus groups were used to elicit views from sub-sections of the UK population about the potential use of such cultured red blood cells. We reflect on the appropriateness of that methodology in the context of the BloodPharma project. Findings are in the form of lessons transferable to other interdisciplinary, science-led teams about what a social science dimension can bring; why qualitative research should be included; and how it can be used effectively.

**Discussion:**

Qualitative data collection offers the strength of exploring ambivalence and investigating the reasons for views, but not necessarily their prevalence in wider society. The inherent value of a qualitative method, such as focus groups, therefore lies in its ability to uncover new information. This contrasts with a quantitative approach to simply ‘measuring’ public opinion on a topic about which participants may have little prior knowledge. We discuss a number of challenges including: appropriate roles for embedded social scientists and the intricacies of doing upstream engagement as well as some of the design issues and limitations associated with the focus group method.

## Introduction

Social scientists have previously studied blood in the contexts of the ‘gift relationship’ [1] and the ‘biovalue’ of blood [2], situated within the wider field of tissue and organ donation and the role of altruism within blood transfusion. While other groups have studied blood replacement technologies [e.g., 3], no previous research has been conducted concerning public attitudes to stem cell derived red blood cells. Working as part of an interdisciplinary research collaboration, involving natural scientists, clinicians and engineers (the ‘BloodPharma project’ [4]), we conducted an empirical study using qualitative research methods, from which we derive lessons transferable to other interdisciplinary teams about why, when and how to engage members of the public around willingness and preferences of emerging medical technologies. Our study of cultured red blood cells serves as an illustrative example of innovation projects within the emerging field of regenerative medicine more broadly; a field surrounded by considerable high expectation, and which is regarded as susceptible to translational challenges including, in particular, those related to public perceptions and concerns.

This article reports on the methodology we used in our study. Our purpose here is threefold. First, we argue for adopting qualitative, focus group research as a useful method for engaging purposively sampled groups in the development of biomedical innovations. We reflect on key research design issues and lessons learned from the process that could be transferable to other teams of scientists and clinicians seeking to work with embedded social scientists in order to gauge potential public reactions to new medical technologies.

There is an on-going debate within the social science community about appropriate roles for the social sciences in interdisciplinary, science-led collaborations, with increasing calls for social researchers to be involved in the earliest, problem-framing and agenda-setting phases of such research [5]. Our second purpose is to extend this debate to biomedical and other natural scientists, whom we anticipate will form the primary audience for this paper.

Thirdly, having witnessed the movement over the past 20 years from education, to participation, and now to much earlier public consultation over emerging science and technology, as methods of increasing the publics’ trust in science, our findings also underscore the challenges of doing this so-called ‘upstream engagement’ [6] in such fields. The BloodPharma team is carrying out upstream engagement well before the cultured blood product is expected to reach the clinic (potentially 20 years from now). Upstream engagement presents a significant challenge to the BloodPharma team, due to its unique position in already having the viable alternative of blood donation. Unlike other entirely new technologies being developed using regenerative medicine, which may provide a unique benefit, the BloodPharma product is a reformulation of an existing technology—blood from donors. The public is therefore not being asked to accept a new product but to choose between the existing (trusted and familiar) blood from human donation and the new BloodPharma product. This presents particular difficulties in explaining why this change is necessary: as one of our interviewees put it, they are ‘creating a demand, not filling a need’.

## Background

Blood donations are now widely used in Western medical care to overcome blood loss through injury, during surgery, or for those conditions that result in severe anaemia [7]. Currently, this blood supply is dependent on many human blood donors. The UK is fortunate in possessing an established donation system, although fluctuations in donor numbers do occur, and even in established blood donation systems there is the potential for transfusion-transmitted infections (TTIs). The BloodPharma project is therefore developing an alternative method of producing red blood cells (RBCs) for use in human transfusion, using stem cell technology, with the eventual aim of producing an unlimited, and infection-free, supply of O rhesus negative (the universal donor) RBCs. Previous attempts by other research teams to culture blood from other means (e.g., bone marrow and cord blood) have not proved successful [8].

Development of a novel method of obtaining red blood cells for transfusion cannot become a substitute for conventional blood donation unless the factors determining uptake are understood. Trust is seen to have a direct impact on the acceptance of technologies, which in turn impacts on how many people are willing to use such technologies and on their potential market value [9, 10]. The EuroBloodSubstitutes project [3] represents one of the few examinations of public attitudes to the use of blood replacement technologies but did not include stem cell derived technology. Although the findings from the EuroBloodSubsitutes study demonstrated that alternatives to donated blood were perceived as ‘more risky’ by the public, there is little basis on which to judge how the public may react to the use of cultured red blood cells from stem cells. Our research therefore sought to elicit the views of a wide variety of publics towards the potential use of cultured RBCs for transfusion. As a result, we were able to offer the BloodPharma team specific recommendations regarding their future interactions with the public and potential users of the BloodPharma product [11].

## Reasons for eliciting public views

A key driver of the BloodPharma outreach is to engage members of the public early in the innovation of the cultured blood product, given evidence [e.g., 12–15] that suggests that patient and clinician views can impact on the uptake of new therapies. There are several reasons for engaging wider publics in the development of scientific research, which include widening expertise, developing information resources, social responsibility, risk management, and gaining public acceptance. For many projects, including the BloodPharma project, the motivations for undertaking public engagement may touch on all of these reasons.

Studies have demonstrated public unease that scientists lack regulatory oversight, and highlight the role that an understanding of regulation can play in building trust between publics and researchers in the area of stem cell technology [e.g., 10]. This taps into wider calls for scientists to step down from the ‘ivory tower’ [16] and attempts to bring engagement with lay audiences ‘upstream’ in order to increase public trust in science [6]. The broader context for this spans both the long-standing science and technology studies literature, which understands innovation to be a complex, interactive process rather than one that follows a linear model (see, for example, [17]), and the more recent discourse around ‘Responsible Research and Innovation’ (launched by the European Commission as part of the Horizon 2020 programme [18]), which argues that innovation needs to be attentive to, and directed towards, the values and perspectives of the public.

The criteria for obtaining research funding also often force researchers to identify at an extremely early stage aspects such as target markets, potential patient groups, specific details about the eventual product, etc. It is recognised that such expectations are crucial for mobilising funding and that these expectations link technical and social considerations [19].

One reason for engaging the views of those outside the BloodPharma project was to tap into the wealth of expertise held by different groups. As we explain below, we sought to include a range of what might conventionally be regarded as ‘lay’ and ‘expert’ views in our data collection. This, for example, included patient groups who may be experts at living with a blood disorder and ongoing treatment. Such individuals may not consider themselves to have formal expertise but the value of such lay knowledge is increasingly recognised [e.g., 20].

There are, however, differences of opinion on the extent to which ‘the public’ should be involved in decision-making regarding the implementation of new technology. Stem cell therapies are not without risks, such as possible contamination and the formation of teratomas [21]. Each individual has their own definition of acceptable risk, resulting in ‘risk cultures’ [22]. The potential risks in the stem cell field have been characterised into ‘individual risks’ (such as the potential for tumour formation or stimulation of latent viruses) and ‘community risks’ (such as the transfer of animal viruses to the human population through the stem cell manufacture) [23]. This leads to debates about the role of ethical or moral values in the risk analysis of new technologies, and concerns that this might override the use of unbiased scientific assessment [24–27]. Upstream engagement may inevitably result in the restriction of some scientific innovations because debate must occur before all the evidence is available [28].

The introduction of new drugs and therapies involves not just the granting of a marketing authorisation but also an element of public willingness to accept the new therapy. In places such as the UK, a decision must be made about whether a drug or therapy is acceptable for use in NHS clinics and hospitals, based largely (but not solely) on economic factors. There is increasing evidence that public acceptance plays an important role in the delivery of a new technology to market [29]; differing uptake rates of new therapies in different countries cannot be explained by health spending alone [30] and many decisions on implementing new therapies fall to clinicians, either at individual or at health board level [31].

Public opinion is an important consideration when developing new stem cell therapies as the potential uptake of the product or therapy can be greatly influenced by the attitudes of the end user (be they the recipient of the RBCs, the clinician, or NHS procurement). However, gauging that public opinion, for example through surveys, can be problematic in areas where there is considerable uncertainty either because the subject matter is quite technical or because its likely application is not yet certain. Focus groups are an especially useful method for generating discussion and exchange of views in this context when the topic is not something that might be widely discussed or known about. They are, nevertheless, not unproblematic in terms of how the data generated embody the differences between opinion, views and beliefs and how this relates to what ‘counts’ as evidence, experience and lay expertise.

## Methods

Our research adopted a qualitative method which is often characterised by its inductive relationship between data and theory: data are used to derive theory, in contrast to the hypothesis-testing approach more commonly favoured by quantitative research methods [32]. Our approach does not claim to discover the views of the whole ‘public’ but to produce in-depth results about a small number of groups.

Our research brought together groups of people who were not targeted for any specific expertise in science, and who represented a range of ages, level of educational attainment, geographical location, etc. We used purposive rather than probabilistic sampling^1^ so, although we attempted to gather data from a wide variety of demographic groups, these results cannot be extrapolated to cover the whole population of the UK.

Four core groups were identified and data collection was carried out with members of these groups across 27 separate sessions (15 individual interviews and 12 focus groups):

- Patients who were regular users of donated blood undergoing multiple transfusions for the treatment of conditions such as thalassaemia and myelodysplasia (MDS)
- Representatives of religious and moral groups who could contribute to the discussion of issues regarding cultured red blood cells from a religious or ethical standpoint
- Clinical groups including nurses and doctors who have the potential to use this blood in the course of their clinical work
- Community groups chosen to represent a spread of publics, without necessarily having any background in science or healthcare. These were recruited through sports club, craft groups, etc., and were an attempt to represent, as far as possible, the ‘general public’.

In this study we used interviews for targeted participants, chosen because they had a particular knowledge base. These included representatives of religious organisations, academics, and doctors. Interviews were also conducted with patients who were too geographically spread, or not in good enough health, to attend a focus group.

Focus groups took a variety of formats. Our standard focus group lasted approximately 2 h. This incorporated a time for giving information about the project, for discussion, and for activities, as well as a break for refreshments. In many of the focus groups, we asked participants to write down words that they associated with blood as a prompt for further discussion. Whilst this was the desired format of the group it was not always possible to recruit participants for 2-h sessions and, in some cases, they were truncated to fit the time available by, for example, omitting the writing activity. In a small number of cases, we had to adapt to circumstances and conduct focus groups with a smaller number of participants for durations of just a few minutes each. Focus groups took place at a variety of public locations, including meeting rooms in local council venues, university rooms, a residential care home for the elderly, and a further education college.

In a focus group, the researcher’s role is to act, not as an interviewer, but as a moderator of the discussion between participants. Focus group schedules (which provide an ‘aide memoire’) for this project were designed following preliminary scoping conversations with the wider project team, an initial review of the literature, and on the results of a small number of pilot interviews. This prepares the researcher with a list of questions or themes to guide the discussion (known as ‘probes’), whilst allowing freedom to change the order of topics, insert new ones, or abandon some—depending on how the conversation progresses.

Consent procedures for participants followed the guidelines of the School of Social and Political Science, University of Edinburgh [34]. The majority of participants were consented using signed consent forms before data collection took place. Where this was not appropriate (for example, the focus groups with parents at a toddler group) then verbal consent was obtained.

All interviews and focus groups were recorded then transcribed verbatim^2^ but with the removal of filler words (‘like’, ‘actually’), non-verbal utterances (‘ummm’, ‘hmmm’) and general chat, where these were not deemed to add anything to the data. To protect confidentiality, pseudonyms were used to identify participants in all transcripts. Data analysis for this project then followed a grounded theory methodology [35], which allows for data collection to take place iteratively, with earlier findings contributing to questions asked during future data collection.

The identification of key themes in qualitative data analysis is a process of pattern recognition and data reduction. ‘Coding’ is a process of identifying these key themes as they occur in the data, in this case in transcripts of interviews and focus groups, and was undertaken using NVivo software. The key themes chosen for coding were identified at the outset of the project (by the team and through the research questions) and augmented by themes that arose during the preliminary interviews and further data collection.

As reflexive social scientists, we then undertook a process of reflection and discussion among the research team and with other colleagues in order to consider both our methods and the results they had produced. This enabled us to assess the outcomes from our engagement in this biomedical project from the perspectives of, inter alia, any disparities between our initial expectations and eventual outcomes, the logistical challenges of undertaking this type of research, and our own experiences as social scientists working as part of a predominantly biomedical team. From this we were able to derive a series of lessons about the design and conduct of such research that we believe would have utility in future interdisciplinary, biomedical innovation projects. Our intention in doing so is not to offer a checklist or protocol for qualitative research methods as others have done (e.g. [36]) but to discuss some of the motivations, challenges and benefits of such an approach in the context of interdisciplinary research teams in order to ensure the appropriate involvement of social science expertise [5].

## Research design decisions

### Choosing interviews or focus groups

Both interviews and focus groups are qualitative research methods that are designed to extract in-depth information from participants. They are similar in the potential topics covered, the process of consent, and the methods of recruitment. Interviews are conducted with one, or sometimes two, participants. The researcher takes the position of interviewer, and asks questions and elicits responses from the participant (interviewee). Interviews can be structured, semi-structured, or unstructured in design [33, 37]. Focus groups are carried out with a larger number of people, ideally four to six participants. The aim of a focus group is to study the discussions within the group, and the researcher is considered to be the moderator or facilitator, rather than an interviewer [38, 39]. The moderator will pose questions and generally chair the discussion, but the aim is to generate discussion among participants, rather than a back and forth dialogue between participants and moderator.

A key research design decision that must be made in a study of this type is whether it is more appropriate to use interviews or focus groups as the main data collection method. In searching the literature (e.g. [40]) and asking advice of colleagues, there were three key considerations that determined our choice of method:

- interviews are more appropriate for participants who have a pre-existing knowledge base about the topic being researched whereas focus groups are better for people who may not have prior knowledge of the topic
- interviews are generally easier to recruit and plan as it only involves the coordination of the participant and the researcher
- interviews offer higher levels of anonymity compared with focus groups. Although consent procedures are fully adhered to for both methods, there is the additional factor of the other participants present at a focus group

### Choosing participants

These three points therefore determined the method that we selected for different participants, for example:

- we used interviews for ‘expert’ participants, e.g., the representatives of religious organisations, academics, doctors
- we used interviews for people whom we found it hard to recruit due to busy schedules, e.g., haematologists
- we used interviews with patients who were scattered around the country and not part of an existing patient group
- we used focus groups for ‘lay’ participants—such as the groups recruited through sports clubs, students, parent and toddler groups
- we used focus groups where we could bring together existing groups, e.g., patient groups.

The aim of the data collection was not to achieve a representative sample of the entire UK population,^3^ but instead to concentrate on obtaining the views of as wide a range of groups as possible. In order to achieve this, we attempted to obtain a representation of both rural and urban populations, a variety of ages, variation in educational attainment, etc.^4^ Some participant groups were targeted due to the perceived information that they could bring to the discussion, for example patients and religious groups.^5^ Other groups were considered representative of the ‘general’ population—for example those focus groups conducted with sports clubs. We had hoped to engage more religious groups, and groups of international students but this was hindered by the lack of gate-keepers who were willing to facilitate access for us. Middle-aged men and blue-collar workers are also notably under-represented in our sample.

### Known or unknown groups

A consideration in focus group recruitment is whether to use groups of individuals who are already known to each other (i.e., social groups) or whether to convene groups of individuals who have not previously met. Known (pre-existing) groups are likely to require fewer ice-breaking exercises as the participants are already known to each other. This can help to put people at their ease. The downside of using known groups is considered to be the presence of already established hierarchies and relationships within the group. In discussions about personal or contentious topics, pre-existing groups may suppress conversations if people do not want to reveal things to people already known to them—and whom they will see after the focus group. Pre-existing groups are also considered easier to recruit. Unknown groups may encourage more openness for certain people or topics, but are considered more difficult to recruit.

In this research we primarily used pre-existing groups. This was due to the nature of the organisations who acted as gate-keepers for us (for example, patient groups) and for speed of recruitment. It was felt that the nature of the topic being discussed did not prevent participants from sharing information with the group, and that the reliability of recruiting established groups outweighed any potential negatives. Some groups (such as the medical students) had participants who were not known personally to each other, but who studied together at the same medical school. Our only ‘unknown’ group resulted in only one participant attending, giving weight to the idea that organising groups of people who are known to each other provides more of a social obligation for people to attend.

### Reporting data

Focus groups analyse the interactions between participants, and every effort should be made to represent this in data analysis [32, 42]. This means that conversations between participants are analysed and reported, and the use of single quotations from the transcript is avoided.

In reporting qualitative data, key themes are identified by the researcher, but are taken from the data gathered during interviews and focus groups. This means there may be factual inaccuracies present, but these are a representation of what participants believe to be true. Additionally, as is common when conducting a range of different focus groups, many of the responses might be considered rather contradictory (for example, in our dataset, participants were against the commercialisation of blood product, whilst recognising the needs of commercial companies in producing pharmaceuticals; similarly, one respondent was accepting of the use of hESC in research into IVF, but against hESC use for other research purposes).

## Lessons learned and recommendations

In many respects, our experience of working in the BloodPharma partnership reflects one of the classic interdisciplinary challenges when an already established natural science project decides to incorporate a social science element (e.g., [43]). In such circumstances, social scientists are often brought into ask the ‘how’ questions rather than the ‘why’ questions: in this case, ‘how will the public respond?’.

This approach risks reducing the social science input to a service or subordination role [43] where the social researchers provide specific, well-defined inputs (such as ‘public engagement’) to another domain without the need for significant interdisciplinary interaction or contribution to advance their own core disciplinary knowledge. In such cases, social scientists may feel they are regarded as mediators rather than as scholars in their own right [44].

The often idealised accounts of cross-disciplinary interaction (criticised in [45]) do not necessarily prepare scientists and clinicians well for the experience of interacting with social researchers: both research funders and project leaders need to set aside time and resources at the outset of such collaborations to ensure maximum value from these interactions [46, 47].

The second lesson we would proffer is to ‘expect the unexpected’ and not to assume that public consultation will only confirm what you already know. At the outset of this study, we had anticipated that the use of embryonic stem cells in the early stages of the research would be a barrier to public acceptance of the cultured blood product. However, this technology was acceptable for the majority of people to whom we spoke, whereas it was the commercial aspect of the BloodPharma project, which might alter the nature of blood from a ‘gift’ to a commercial commodity, that garnered the most concerns [11]. This reinforces the added value of a qualitative method, such as focus groups, in terms of generating new information, rather than a quantitative approach to simply ‘measuring’ public opinion, on a topic about which participants may have little prior knowledge. Nevertheless, there are limitations with this method, most notably the challenge of translating data into policy and practice when the sampling may be purposive rather than statistically representative of whole populations.

Thirdly, issues of informed consent will always be a major consideration in research of this type. We had opted to obtain Level 2 ethical consent from the University of Edinburgh as we sought to engage with participants who had particular medical conditions. However, we decided not to recruit through clinics, in part because this would have required the more stringent (and time-consuming) NHS ethics approval but also, significantly, because we did not want to involve patients who were under pressure during times of illness. For our particular research project, we were steered away from recruiting participants from blood donation sessions, (which would have been an obvious gateway), due to sensitivities on the part of the blood transfusion service surrounding any unintended impressions that donors might gain regarding any potential future reduction in the need for donors. Similar research in other areas of new medical technology might present their own unique sets of circumstances, intensifying the challenges of obtaining ethical approval.

Our fourth set of lessons relates to access and design choices, summarised in Table 1. Given that we had opted for a greater use of ‘known groups’ in our recruitment strategy, we relied heavily on the co-operation of various gatekeepers to provide access to potential participants but we encountered difficulties with recruitment beyond what we had anticipated at the start of the project. This led to a number of groups whom we had hoped to engage being dropped from our data collection within the timeframe of our project. The lesson we draw from this is to plan for longer lead times and to consider the pros and cons of identifying key individuals who may be able to facilitate introductions to such groups.

**Table 1:** Research design choices when eliciting public views

Research design context	Preferred method
The informant has specialist knowledge of the topic	One-to-one interview
The informant is likely to have less technical knowledge of the topic	Focus group
The informant is likely to be inhibited by the presence of people whom they already know	Unknown focus group
Access and recruitment are likely to be difficult	Known focus group

Many of the patients were recruited through gatekeeping organisations such as patient groups. It became apparent that many of these groups are run by resource- and time-limited volunteers and our request was very far down their priority list. In one case, it took over a year from first contact to the gatekeeper putting us in touch with potential patients. Some of the charities also declined to let us access or post anything on Facebook or blog pages, and insisted that our project proposal, and the work of the BloodPharma project, were reviewed by their own medical advisers.

Many groups simply failed to respond to any contact made, despite numerous reminders from the researchers. We also encountered a number of gatekeepers who were unfamiliar with social science data collection methods. These included academic departments such as medical schools that required us to obtain their own ethical consent, the wording of which was not applicable for social science data collection methods.

Difficulty in obtaining participants for social science research is not unusual, although this normally happens at the level of the actual focus groups. For example, the literature normally recommends over-recruiting [48], to allow for people not attending at the allocated time. Our experience was that we had no last-minute non-attendance at the groups we had organised (other than the group of unknowns, reinforcing what has already been established in the literature about the difficulty of recruiting such groups), but that the original organisation of these groups through the gatekeepers was the main barrier to data collection.

Finally, there are broader problems in applying upstream engagement to new medical technologies: conflict may arise from a complex mixture of uncertainty, power politics, divergent societal interests, values and ideologies, and commercial competition. Upstream engagement can create opportunities to reinforce the negative views of new technologies where, as we have discussed elsewhere [49], there may be no societal consensus about whether we should develop particular technologies. Engagement should be carefully timed: too early (‘upstream’) and its value will be undermined by uncertainty about the nature of future developments; too late and stakeholder opinions and political positions may have become entrenched so that accommodation will be more difficult to achieve.

## Conclusions

We have demonstrated that qualitative research and, specifically, focus-group studies have a key role as part of interdisciplinary, biomedical innovation projects. We have identified generalisable lessons that will further the clinical research community’s ability to enlist robust methodologies for identifying the public’s willingness and preference for emerging medical technologies. Specifically, this study enabled people to ask questions about the BloodPharma project and to voice any concerns. Data gained from these focus groups will contribute to the design and delivery of project information, for example, through the BloodPharma (now Novosang) website.

Notably, our difficulty in recruitment speaks to a wider question about why we should expect the public to engage in medical and scientific research, and to give up their time in order to do so. This is echoed in the public engagement literature [e.g., 50] which is moving from an emphasis on engaging the public to a more reflective look at why and how the public should be engaged, and a growing recognition of why the public may (or may not) want to engage with science.

Nevertheless, our experience offers a number of practical lessons regarding the design and conduct of such research that should help teams of scientists and clinicians maximise the value of future engagements with public audiences through social science research.

## Footnotes

1 Readers unfamiliar with social research methods may find a text such as Bryman [33] helpful if further definitions are required.

2 The only exception to this was a small number of focus groups where handwritten notes were taken.

3 Due the nature and funding of the BloodPharma project, data collection was focused within Scotland. In one case (the Thalassaemia group), it was not possible to obtain a large enough number of patients in Scotland, and so this took place in England.

4 It was not possible for demographic information, including gender and age, to be taken at every focus group but information that was collected showed a slightly higher proportion of males to females; participants’ ages ranged from 18 to 80+.

5 It was decided to limit the involvement of GPs and other health service workers as their views had already been sought in a previous report commissioned by the BloodPharma project [41].
